# Metacognitive ability correlates with hippocampal and prefrontal microstructure

**DOI:** 10.1016/j.neuroimage.2017.02.008

**Published:** 2017-04-01

**Authors:** Micah Allen, James C. Glen, Daniel Müllensiefen, Dietrich Samuel Schwarzkopf, Francesca Fardo, Darya Frank, Martina F. Callaghan, Geraint Rees

**Affiliations:** a Institute of Cognitive Neuroscience, UCL, UK; b Wellcome Trust Centre for Neuroimaging at UCL, UK; c Department of Psychology, Goldsmiths, University of London, UK; d Experimental Psychology UCL, 26 Bedford Way, WC1H 0AP London, UK; e Danish Pain Research Centre, Department of Clinical Medicine, Aarhus University, 8000 Aarhus, Denmark; f Interacting Minds Centre, Aarhus University, 8000 Aarhus, Denmark; g Division of Neuroscience and Experimental Psychology, University of Manchester, UK

**Keywords:** Metacognition, Quantitative MRI, Hippocampus, Iron, Myelination, Microstructure

## Abstract

The ability to introspectively evaluate our experiences to form accurate metacognitive beliefs, or insight, is an essential component of decision-making. Previous research suggests individuals vary substantially in their level of insight, and that this variation is related to brain volume and function, particularly in the anterior prefrontal cortex (aPFC). However, the neurobiological mechanisms underlying these effects are unclear, as qualitative, macroscopic measures such as brain volume can be related to a variety of microstructural features. Here we leverage a high-resolution (800 µm isotropic) multi-parameter mapping technique in 48 healthy individuals to delineate quantitative markers of *in vivo* histological features underlying metacognitive ability. Specifically, we examined how neuroimaging markers of local grey matter myelination and iron content relate to insight as measured by a signal-theoretic model of subjective confidence. Our results revealed a pattern of microstructural correlates of perceptual metacognition in the aPFC, precuneus, hippocampus, and visual cortices. In particular, we extend previous volumetric findings to show that right aPFC myeloarchitecture positively relates to metacognitive insight. In contrast, decreased myelination in the left hippocampus correlated with better metacognitive insight. These results highlight the ability of quantitative neuroimaging to reveal novel brain-behaviour correlates and may motivate future research on their environmental and developmental underpinnings.

## Highlights

• Metacognitive ability (insight) differs strongly between individuals.
• We used quantitative MRI to relate metacognition to brain microstructure.
• Insight correlated positively with anterior prefrontal myelination.
• Myelination of the left hippocampus negatively related to insight.
• Iron levels in the visual cortex were negatively associated with metacognition.

## Introduction

The metacognitive capacity for self-monitoring is at the core of learning and decision-making (Flavell, 1979). As a general capacity, metacognition is thought to enable the flexible monitoring and control of perception, memory, and other cognitive processes (Fernandez-Duque et al., 2000). An efficient approach to quantifying this ability lies in the application of signal-detection theory to estimate the sensitivity of self-reported confidence to objective discrimination performance (Fleming and Lau, 2014). Individual differences in metacognitive sensitivity thus quantified are related to the morphology, function, and connectivity of the anterior prefrontal cortex (aPFC), precuneus, and other cortical areas (Fleming and Dolan, 2012). Here, we expand on these findings using a recently developed multi-parameter mapping (MPM) and voxel-based quantification (VBQ) technique to better elucidate the neurobiological mechanisms underpinning these effects.

The volume and function of the anterior prefrontal cortex (aPFC) and precuneus have repeatedly been related to metacognitive ability (Fleming et al., 2014, Fleming et al., 2012, Fleming et al., 2010a, McCurdy et al., 2013, Sinanaj et al., 2015). Notably, several studies found a positive relationship between right aPFC volume and metacognition (Fleming et al., 2010a, McCurdy et al., 2013, Sinanaj et al., 2015). While convergent evidence from anatomical, lesion-based, and functional connectivity studies suggest that the right aPFC is specific to perceptual metacognition, metacognition for memory has instead been related to midline cortical (e.g., mPFC and PCC/precuneus) and hippocampal structures (Baird et al., 2013, Fleming et al., 2014, Fleming et al., 2012, McCurdy et al., 2013). Although these studies suggest that the ability to introspect on perception and memory depends on the development of a neural mechanism involving both domain-specific and general aspects, the underlying neurobiology driving the relationship between neuroanatomy and metacognition remains unclear.

One important source of uncertainty is the inherent lack of specificity offered by volumetric measures of brain structure, which are fundamentally qualitative in nature. Indeed, voxel-based morphometry (VBM) yields measures in arbitrary units which can be driven by a variety of macroscopic factors such as cortical thickness and variability in cortical folding, owing to a non-specific variety of microstructural features (Ashburner, 2009). It has recently been shown that microstructural properties of brain tissue, such as myelination levels and iron content can lead to the detection of spurious morphological changes (Lorio et al., 2016, Lorio et al., 2014). As volumetric measures are highly dependent on acquisition parameters which can vary widely across scanners, field strengths, and other variables, they are difficult to directly replicate across studies (Boekel et al., 2015).

The emerging field of *in vivo* histology aims to address these issues by combining maps of specific MRI parameters measured via quantitative imaging (qMRI) with biophysical models and voxel-based quantification (VBQ) techniques to provide direct indicators of the microstructural mechanisms driving morphological findings. This approach quantifies biologically relevant metrics such as myelination and iron concentrations, oligodendrocyte distributions, and the g-ratio of fibre pathways (Mohammadi et al., 2015, Weiskopf et al., 2015). As these measures are quantitative in nature, they are largely invariant to specific scanner protocols and offer improved neurobiological specificity, thus facilitating our understanding of brain-behaviour mechanisms, improving reproducibility, and identifying novel biomarkers for clinical research.

In the present study, we used qMRI to map a number of key contrast parameters with differential sensitivity to underlying biological metrics, in order to better understand the microstructural correlates of metacognitive ability. To do so, we acquired high-resolution (800 µm isotropic) data using the Multi-Parametric Mapping (MPM) qMRI protocol (Weiskopf et al., 2013). We then conducted voxel-based quantification (VBQ) analysis (Draganski et al., 2011) in 48 healthy participants to relate these microstructural markers to individual differences in metacognitive sensitivity during an adaptive visual motion discrimination task. Our results revealed that right aPFC markers of myeloarchitecture (increased R_1_ & MT) positively correlate with metacognitive ability, whereas the dentate gyrus of the left hippocampus showed effects consistent with decreased myelination (reduced MT). Cortical iron markers in the precuneus (increased R_2_*) and visual cortex (decreased R_2_*) also covaried with metacognitive ability. These results extend our understanding of the microstructural neuroanatomy of metacognition and provide novel targets for future clinical research.

## Methods

### Participants

48 healthy participants (29 female) were recruited from University College London and the surrounding community. As age is a strong determinant of brain microstructure (Callaghan et al., 2014), we restricted our inclusion criteria to 20–40 years, resulting in a mean age of 24 (SD = 5). All participants were right handed, and were mentally and physically healthy with no history of neurological disorders and with normal (or corrected-to normal) vision and hearing. Participants were recruited from a local participant database using broadcast emails. All participants gave informed written consent to all procedures. In accordance with the Declaration of Helsinki, the University College London Research Ethics Committee approved all procedures.

### Study design

Participants completed the experiment in two sessions, consisting of a 2-h appointment at the Wellcome Trust Centre for Neuroimaging to acquire all imaging data, and a separate 1-h appointment to complete the metacognition task, a brief non-verbal auditory memory measure (Harrison et al., 2016, Müllensiefen et al., 2014), a tonotopy functional scan, and other auditory and behavioural measures as part of a study on individual differences in the auditory cortex (data not reported here). The neuroimaging session involved 30 min of multi-parameter mapping (MPM) while subjects silently viewed a muted nature documentary to maintain wakefulness (and hence limit motion). During the behavioural session, participants completed an adaptive psychophysical visual metacognition task (see *Behaviour*, below) lasting 30 min.

### Behaviour – Metacognition Global Motion Task

To measure participants' metacognitive ability, we employed a global dot motion discrimination task comprising a forced-choice motion judgement with retrospective confidence ratings on every trial. As part of another investigation, in which we were investigating noise-induced confidence bias (Spence et al., 2016; Allen et al., 2016), we used a dual-staircase approach with two conditions in which either mean direction or standard deviation across dot directions was continuously adapted to stabilize discrimination performance. Thus, to control sensory noise independently of task difficulty, in two randomly interleaved conditions we presented either a stimulus with a fixed 15-degree mean angle of motion from vertical and a variable (adaptive) standard deviation (SD), or a variable (adaptive) mean angle from vertical at a fixed 30° SD. In either case, the mean (μ-staircase condition, μ*S*) or standard deviation (σ-staircase condition, σ*S*) of motion was continuously adjusted according to a 2-up-1 down staircase, which converges on 71% performance. On each trial the motion signal was thus constructed using the formula:

$DotDirections=\left(Left\;|\;Right\right)\;\times \;MeanOrientation\;\;+\;\left(GaussianNoise\;\times \;SD\right)$

In which the condition-specific staircase determined either mean orientation or SD. Each trial consisted of a 500 ms fixation, followed by a 250 ms central presentation of the motion stimulus, which was then replaced by a central letter display “L R”. Participants then had 800 ms to make their response to indicate whether the mean motion direction was to the left or right of vertical. After this, a confidence rating scale marked by 4 equal vertical lines appeared. Each line was labelled, from left to right “no confidence, low, moderate, high confidence”. Participants’ heads were fixed with a chin and forehead rest 72 cm from the screen. Motion stimuli consisted of a central array of 1100 dots presented over a central fixation dot, within a circular aperture of radius 9.5° visual angle (DVA), with dots advancing 0.02 DVA per frame. To ensure participants attended the global rather than the local motion direction, dot lifetimes were randomized and limited to a maximum of 93% stimulus duration.

**Fig. 1:**
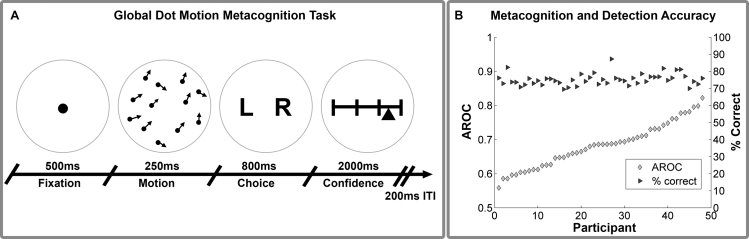
Behavioural Paradigm and Metacognitive Accuracy. Schematic of global dot-motion metacognition task (A) and plot of metacognitive vs motion detection accuracy (B). Participants were required to judge the global or average motion of a brief dot display, and then rate their confidence in this judgement from 0 (guessing) to 100 (certain). Performance was held constant using an adaptive threshold adjusting either signal mean or variance on each trial (see *Methods* for more details). Right hand plot demonstrates substantial individual differences in metacognitive accuracy, estimated as the type-II area under the curve (AROC), independently of motion discrimination performance. Inter-individual differences in AROC were then used in a multiple regression analysis to explain variation in microstructural brain features (see *VBQ Analysis*).

Participants were instructed that the goal of the task was to measure their perceptual and metacognitive ability. Metacognitive ability was defined as a participant's insight into the correctness of their motion judgements, i.e. how well their confidence reports reflected their discrimination accuracy. Participants completed a short practice block of 56 trials, in which they performed the motion discrimination without confidence ratings, with choice accuracy feedback provided by changing the colour of the fixation to green or red. All participants achieved better than 70% accuracy and indicated full understanding of the task before continuing. Participants completed 320 trials divided evenly between the two staircase conditions. Trials were divided into 10 blocks each with 40 trials, randomly interleaved across conditions within each block. 14 participants did not complete the last two blocks of the task due to a technical error, however all participants had at least 100 trials per condition (Fleming and Lau, 2014). See Fig.1 for an graphical summary of our task.

### Quantitative Multi-Parameter Mapping (MPM)

Recent technical developments have enabled *in vivo* mapping of neuroimaging markers of biologically relevant quantities to be performed with high resolution and whole brain coverage (Deoni et al., 2005, Helms et al., 2009, Helms et al., 2008a, Helms et al., 2008b). We used the Multi-Parameter Mapping (MPM) protocol (Weiskopf et al., 2013) to obtain maps of the percent saturation due to magnetization transfer (MT), longitudinal relaxation rate (R_1_), and effective transverse relaxation rate (R_2_*).

### Data acquisition

All imaging data were collected on a 3T whole body MR system (Magnetom TIM Trio, Siemens Healthcare, Erlangen, Germany) using the body coil for radio-frequency (RF) transmission and a standard 32-channel RF head coil for reception. A whole-brain quantitative MPM protocol consisting of 3 spoiled multi-echo 3D fast low angle shot (FLASH) acquisitions with 800 µm isotropic resolution and 2 additional calibration sequences to correct for inhomogeneities in the RF transmit field (Callaghan et al., 2015b, Lutti et al., 2012, Lutti et al., 2010).

The FLASH acquisitions had predominantly proton density (PD), T1 or MT weighting. The flip angle was 6° for the PD- and MT-weighted volumes and 21° for the T1 weighted acquisition. MT-weighting was achieved through the application of a Gaussian RF pulse 2 kHz off resonance with 4 ms duration and a nominal flip angle of 220°. The field of view was 256mm head-foot, 224 mm anterior-posterior (AP), and 179 mm right-left (RL). Gradient echoes were acquired with alternating readout gradient polarity at eight equidistant echo times ranging from 2.34 to 18.44 ms in steps of 2.30 ms using a readout bandwidth of 488 Hz/pixel. Only six echoes were acquired for the MT-weighted acquisition in order to maintain a repetition time (TR) of 25 ms for all FLASH volumes. To accelerate the data acquisition, partially parallel imaging using the GRAPPA algorithm was employed with a speed-up factor of 2 in each phase-encoded direction (AP and RL) with forty integrated reference lines.

To maximise the accuracy of the measurements, inhomogeneity in the transmit field was mapped using the 2D STEAM approach described in Lutti et al. (2010), including correcting for geometric distortions of the EPI data due to B0 field inhomogeneity. Total acquisition time for all MRI scans was less than 30 min.

### Parameter Map Estimation and Voxel-Based Quantification (VBQ)

All images were processed using SPM12 (version 12.2, Wellcome Trust Centre for Neuroimaging, http://www.fil.ion.ucl.ac.uk/spm/) and bespoke tools implemented in the voxel-based quantification (VBQ) toolbox version 2e (Draganski et al., 2011, Weiskopf et al., 2015), implemented in MATLAB (Mathworks Inc, version R2014a).

To create the quantitative maps, all weighted volumes were co-registered to address inter-scan motion. Maps of R_2_* were estimated from the gradient echoes of all contrasts using the ordinary least squares ESTATICS approach (Weiskopf et al., 2014). The image data for each acquired weighting (PDw, T1w, MTw) were then averaged over the first six echoes to increase the signal-to-noise ratio (SNR) (Helms et al., 2009). The three resulting volumes were used to calculate MT and R_1_ as described in Helms et al., 2008a, Helms et al., 2008b) including corrections for transmit field inhomogeneity and imperfect spoiling (Callaghan et al., 2015c, Preibisch and Deichmann, 2009a, Preibisch and Deichmann, 2009b). The MT map depicts the percentage loss of signal (MT saturation) that results from the application of the off-resonance MT pre-pulse and the dynamics of the magnetization transfer (Helms et al., 2008b).

A Gaussian mixture model implemented within the unified segmentation approach was used to classify MT maps into grey matter (GM), white matter (WM) and cerebrospinal fluid (CSF) (Ashburner and Friston, 2005). Diffeomorphic image registration (DARTEL) was used for spatial non-linear registration of individual grey and white matter tissue classes generated from the structural MT maps to a group mean template image (Ashburner, 2007). The resulting DARTEL template and participant-specific deformation fields were used to register the MT, R_1_ and R_2_* maps of each participant to standard MNI space. We based our non-linear registration of the quantitative map on the MT maps because of their greatly improved contrast in subcortical structures, e.g., basal ganglia, and similar WM/GM contrast in the cortex to T1-weighted images (Helms et al., 2009). A 4 mm full-width at half-maximum (FWHM) Gaussian smoothing kernel was applied to the R_1_, MT, and R_2_* during non-linear registration using the VBQ approach, which aims to minimise partial volume effects and optimally preserve the quantitative values (Draganski et al., 2011). This tissue-specific approach to smoothing generates grey and white matter segments for each map. The grey matter segments were used in all subsequent analyses. For results visualization, an average MT map in standard MNI space was created from all participants.

## Analysis

### Behavioural Analysis – Detection Performance and Metacognitive Ability

All behavioural data were pre-processed using MATLAB (The Mathworks Inc, Natick, MA, USA). Following previous investigations (Fleming et al., 2012, Fleming et al., 2010a), we discarded the first block of trials to allow for staircase stabilization. Any trial with reaction times (RT) below 100ms or more extreme than 3SD of mean RT was rejected from analysis. To quantify metacognitive ability, we estimated the type-II area under the receiver-operating curve (AROC) (Fleming et al., 2012, Fleming et al., 2010a, Fleming and Lau, 2014) separately for each staircase condition. AROC was calculated using the same metric as in Fleming et al., 2010b, Kornbrot, 2006):

(0.1) $$A_{ROC}=\frac{1}{4}\sum_{k=1}^{\frac{1}{2}i}\left({\left[h_{k+1}-f_{k}\right]}^{2}-{\left[h_{k}-f_{k+1}\right]}^{2}\right)+\frac{1}{4}\sum_{k=\frac{1}{2}i}^{i}\left({\left[h_{k+1}-f_{k}\right]}^{2}-{\left[h_{k}-f_{k+1}\right]}^{2}\right)$$

where *i* indicates the four confidence rating bins, *h* depicts the relative frequency of this rating for correct choices ($h_{i}=p(confidence==i|correct)$) and *f* describes the counterpart for incorrect responses ($f_{i}=p(confidence==i|incorrect)$). Under equal performance the AROC thus describes the sensitivity of a participant's confidence ratings relative to their actual performance. For AROC estimation, confidence ratings were binned into 4 equally sized quartiles in MATLAB. As a general index of metacognitive ability, we then calculated average AROC, as well as average confidence, mean accuracy (% correct responses), detection sensitivity (*d’*), choice bias (*c*), and reaction time (*RT*). We also calculated median signal mean, median signal variance, and accuracy within each condition to characterize our thresholding procedure.

### VBQ Analysis – Metacognitive Ability

We initially focused on extending previous volumetric findings relating metacognitive ability to neuroanatomy (Fleming et al., 2014, Fleming et al., 2010a, McCurdy et al., 2013, McCurdy et al., 2013, Sinanaj et al., 2015). To do so we conducted volume of interest (VOI) multiple regression analyses using 5mm-radius spherical VOIs centred on the peak coordinates reported by Fleming (2010a) and McCurdy (2013). This involved the creation of VOIs in the left (−20, 53, 12), (−12, 54, 16) and right (24, 65, 18), (33, 50, 9), (32, 50, 7) aPFC, right dorsolateral PFC (36, 39, 21), and precuneus (6, −57, 18), (8, −64, 24). All VOIs were generated using the WFU Pickatlas Version 3.0.5 (Maldjian et al., 2003), and combined into a single mask. The resulting 5492 voxel (inclusive) mask is available for download at Neurovault.org (http://neurovault.org/collections/1260/).

We then modelled inter-subject variation in the MT, R_2_*, and R_1_ grey matter maps in separate random-effects multiple regression analyses, modelling average AROC as our key dependent variable. Importantly, we followed previous investigations and controlled all analyses for average discrimination sensitivity (d-prime), average confidence, and response bias (*c*) (Fleming et al., 2010a). Additionally, to control for any potential impact of our variance manipulation of metacognitive ability, we included the variance-induced confidence bias, and the difference in mean signal between the two staircases. To estimate the variance-induced confidence bias, we fit multiple regression models within each subject, modelling trial-wise mean, variance, accuracy, and RT as predictors of confidence. This provided beta-weights for each participant indicating the degree to which their confidence report reflected variance-specific bias independently of the other modelled factors, which were then included in our VBQ multiple regression.

Following recommended procedures for computational neuroanatomy, we also included age, gender, and total intracranial volume as nuisance covariates (Ridgway et al., 2008). We then conducted small-volume corrected analyses of the positive and negative main effect of metacognitive ability (AROC) within our *a priori* mask, correcting for multiple comparisons using Gaussian Random Field Theory, FWE-peak corrected alpha = 0.05. Further, we analysed the whole-brain maps of the same contrasts, using a non-stationarity corrected FWE-cluster p-value with a *p*<.001 inclusion threshold (Hupé, 2015, Ridgway et al., 2008). All anatomical labels and percent activations were determined using the SPM Anatomy Toolbox (Eickhoff et al., 2005).

## Results

### Behavioural results

To confirm staircase stability, we first performed two-way repeated measures ANOVA (factor A: block, levels 1–7; Factor B: staircase condition, μ*S vs* σ*S*) on accuracy scores after removing the first block. As several subjects did not complete the last two blocks, we first re-binned trials into 8 equal size bins of 20% total trial length, before analysing block stability. This analysis revealed a significant main effect of variance on accuracy (*F*(1, 47) = 15.15, *p*<.001), but no main effect of block (*ps>*.33) or block by condition interaction (*p*>.11), indicating that although average performance was slightly higher in the σ*S* condition (Mean Accuracy μ*S*=73.4%, Mean Accuracy σ*S* = 76.6%), this difference did not change over time, indicating stable performance within each staircase. As a further check, we repeated this analysis separately within each condition; in both cases the block main effect was not significant (all *ps>*.13). All participants thus achieved stable performance, with an average accuracy of 75.3% (SD = 3%) across the two conditions. Metacognitive ability was comparable to previous studies using the AROC (*M* = 0.68, *SD*=0.06) and did not differ between conditions, *t* (47)=−0.67, *p*=.51. Table 1 presents descriptive statistics for discrimination and metacognition performance.

#### Neuroimaging results

##### VOI analysis – extension of previous aPFC and precuneus findings

**Fig. 2:**
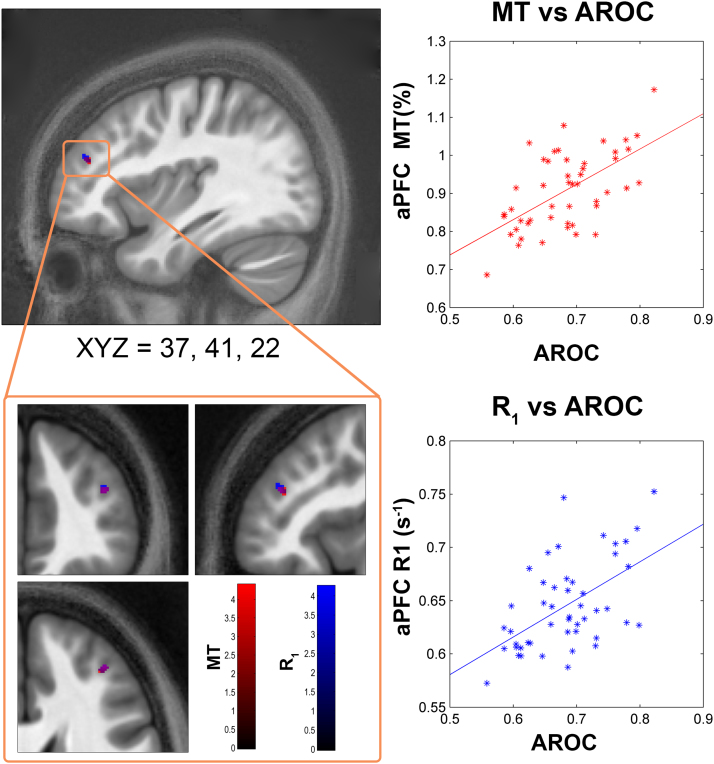
aPFC VBQ findings. Figure shows correlation of metacognitive ability (AROC) and anterior prefrontal (aPFC) microstructural measures of white-matter concentration (R_1_ and MT) across 48 participants. Orientation of crosshairs given below the top left brain in MNI XYZ coordinates. Right side, scatter plots showing peak voxel vs AROC, with least-squares line for illustration purposes. Bottom left, zoomed in view shows overlap of AROC correlation in both MT and R_1_ maps. Colour bars indicate t-values, blobs displayed on average MT map from our 48 participants. Volume of interest analysis, FWE-peak corrected *p*<0.05 within mask generated from previously reported coordinates (Fleming et al., 2010a, McCurdy et al., 2013). See *VBQ Analysis* for more details.

Our VOI analysis revealed significant correlations within the right retrosplenial cortex, precuneus, and aPFC. aPFC showed overlapping, significant positive correlations of AROC with both R_1_ (peak voxel MNI_xyz_ = [37 41 22]) and MT (peak voxel MNI_xyz_ = [37 42 22]) maps. As both maps are sensitive to myelination to a varying degree, this suggests previous volumetric findings in the aPFC are related to the myelo-architecture of the cortical grey matter. We also found that MT in the retrosplenial visual cortex (in a cluster which partially overlaps the precuneus) was negatively related to AROC (peak voxel MNI_xyz_ = [10 −58 8]), whereas R_2_* in the precuneus was positively related to AROC (peak voxel MNI_xyz_ = [9 −64 24]). See Table 2 and Fig. 2 for summary of these results.

### Whole-brain AROC analysis

**Fig. 3:**
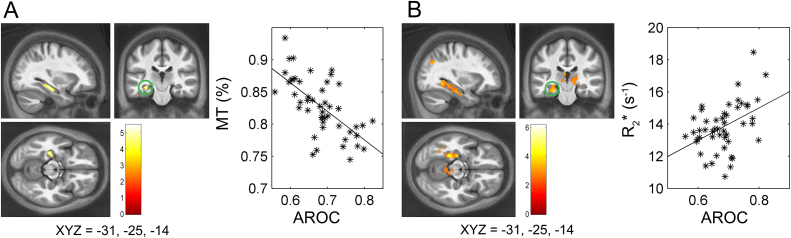
Hippocampal markers of myelination and iron correlate with metacognitive ability. Voxel-based quantification results in the left hippocampus. Hippocampus MT negatively relates to metacognitive ability (AROC) (top left, A), whereas iron levels in the same region positively predict metacognition, albeit at exploratory thresholds only (top right, B). Orientation of crosshairs given below each brain in MNI XYZ coordinates. Blobs depict results of whole-brain multiple regression analyses vs each map type, while controlling for age, gender, ICV, and a variety of performance-related variables (see *Methods* for more information). Results shown on average MT map in MNI space. Scatterplots are for illustration only and depict the peak voxel from each SPM versus raw AROC. Colorbars indicate *t*-values at each voxel. SPMs are FWE-cluster corrected whole brain analysis, *p*FWE < 0.05.

Our whole brain analysis revealed a striking relationship between AROC and left hippocampal MT. Here, higher AROC related to reduced MT in the left posterior-hippocampus (peak voxel MNI_xyz_ = [−31 −25 −14]). Please see Table 2 and Fig. 3 for summary of these results. Inspection of this result in the SPM anatomy toolbox revealed that the majority of the cluster (68.2%) was in the dentate gyrus (43.6% ‘activated’), extending into areas CA1 (4.5%, 2.2% activated), CA2 (3.8%, 7.4% activated), and CA3 (5.1%, 13.2% activated). Additionally, R_2_* correlated negatively with AROC in bilateral visual cortex (52.8% in left V1, 2.0% activated, 35.6% in right V1, 1.3% activated, peak voxel MNI_xyz_ = [1 −69 11]) and positively in the left middle-temporal gyrus (peak voxel MNI_xyz_ = [−51 −48 2]). A positive iron effect which was just above our cluster-level threshold (*p*FWE cluster = 0.052), but which was FWE-peak significant (*p*FWE peak = 0.044, *t* = 6.17) was also present in the right subiculum (peak voxel MNI_xyz_ = [14 −38 −5]).

Finally, as previous studies suggest that decreased MT frequently co-occurs with increased R_2_* and that this inverse pattern may indicate a demyelination mechanism (Steiger et al., 2016), we were interested to see if a similar pattern could be found in the left hippocampus at a reduced (i.e., more exploratory) threshold. We thus lowered our uncorrected inclusion threshold to *p* < 0.005, FWE-cluster corrected to *p* < 0.05, and found that left hippocampal R_2_* (15.0% in left dentate gyrus, 29.% activated, 18.6% in left subiculum, 16.2% activated) also positively predicted AROC (peak voxel MNI_xyz_ = [−26 −36 −13], *p*FWE = 0.011, *t* = 4.94). All raw t-maps and FWE-thresholded maps have been made available to view and freely download at Neurovault (http://neurovault.org/collections/1260/).

### Exploratory analyses

To further evaluate these effects, we performed several post-hoc exploratory analyses, to inform future research on the microstructural underpinnings of metacognition. To this end we assessed 1) whether any of the hippocampus-metacognition correlation was related to a measure of (auditory) memory capability, 2) whether brain iron in other cortical areas was associated with the link between metacognition and hippocampus, 3) whether any volumetric effects underpinned these or our other results, and 4) whether any informative effects could be found in the proton-density maps. Please see Supplementary Methods and Supplementary Figures 1–5 for overview of these analyses and their results.

## Discussion

Our findings demonstrate that individual differences in perceptual insight are related to quantitative markers of cortical myelination and iron. Previous studies investigating individual metacognitive ability indicated that the function, connectivity, and volume of anterior prefrontal cortex (aPFC) underlie introspective accuracy (Baird et al., 2013, Fleming et al., 2010a, McCurdy et al., 2013, Sinanaj et al., 2015). Here we build on these findings using quantitative magnetic resonance imaging to show that metacognition is related to the cortical myelination of the anterior-prefrontal cortex. Further, our hippocampus results suggest that metacognition for perception may require the engagement of both domain-specific and domain-general processes.

Although each of the multi-parameter maps have enhanced specificity over conventional weighted imaging and each exhibit sensitivity to particular microstructural tissue properties, this relationship is not unique. Histologically, differences in MT measures strongly correlate with myelin content in both white (Mottershead et al., 2003, Schmierer et al., 2007, Turati et al., 2015) and gray matter (Fjær et al., 2013). While myelin is also a significant determinant of R_1_ (Gouw et al., 2008, Koenig, 1991, Mottershead et al., 2003), other features of the myeloarchitecture such as the axonal diameter, perhaps coupled to the exposed myelin surface, may be a greater determinant, at least in white matter (Harkins et al., 2016). Nonetheless, other factors such as iron content (Callaghan et al., 2015a, Gelman et al., 2001, Rooney et al., 2007) and cellular architecture (Gouw et al., 2008, Mottershead et al., 2003) also play a role. Similarly, although R_2_* is highly correlated with iron content (Langkammer et al., 2010) it can also be influenced by fibre orientation and architecture (Wharton and Bowtell, 2012). Considering these factors, we benefit from having a multi-modal view to aid in interpreting our findings; the co-localisation of increases in both MT and R_1_ in the aPFC suggests increased myelination independent of changes in iron or other paramagnetic content that would also be expected to impact R_2_*.

We also found that metacognition was negatively related to markers of gray matter myelination (i.e., MT) in the left hippocampus, and positively related to markers of microstructural iron (i.e., R_2_*) in the precuneus, left hippocampus (albeit at exploratory thresholds), and right subiculum. These areas are core nodes of a bilateral memory-related network (Buckner et al., 2011, Choi et al., 2012, Greicius et al., 2009, Yeo et al., 2011), and have previously been implicated in confidence and metacognitive for memory rather than perception (Baird et al., 2013, Chua et al., 2006, McCurdy et al., 2013, Molenberghs et al., 2016). While the hippocampus itself has a central role in visuospatial memory, scene perception, and learning (Hartley et al., 2014, Maguire et al., 2016, Zeidman et al., 2014), as part of the default mode network these areas are also more generally involved in higher cognitive functions involving self-generated thought, episodic memory, visual imagination, and prospection (Andrews-Hanna et al., 2014, Zeidman et al., 2014, Zeidman and Maguire, 2016). Thus although previous functional neuroanatomy studies have tended to emphasize the domain-independence of brain areas involved in meta-memory and perception, these results suggest these areas contribute a domain-general input to metacognition ability. Future work may build on these results by investigating the overlapping and independent microstructural correlates for metacognition in a variety of perceptual and cognitive domains.

It is interesting to further consider the possible neurobiology underlying the negative relationship between hippocampal MT and metacognition. While ex vivo studies suggest that R_2_* is strongly correlated with brain iron (Langkammer et al., 2010), MT is sensitive to macromolecules, with the primary determinant being myelin (Fjær et al., 2013, Schmierer et al., 2007). In general, gray matter myelination is primarily found in local connections between neurons, which bridge between adjacent cortical layers (Hellwig, 1993, Nieuwenhuys et al., 2015). Metacognition requires an accurate mapping between the accumulation of evidence determining perceptual accuracy and subjective confidence (Fetsch et al., 2015, Fleming and Dolan, 2012, Kiani and Shadlen, 2009), which is typically reported retrospectively (and thus depends on accurate and efficient memory). Our finding of reduced hippocampal MT relating to improved metacognition may therefore suggest that a more sparse local connectivity structure in the hippocampus is important for the accurate encoding and/or recall of decision evidence. Although we can only speculate as to the exact mechanism at play here, this result suggests that high resolution imaging in conjunction with dynamic connectivity modelling of hippocampal subfields may further reveal the computational mechanisms which facilitate accurate introspective insight.

### Limitations and future directions

In the present study our paradigm was not designed to tease apart these specific mechanisms; an important future step will therefore be to examine the relationship of brain microstructure and specific decision variables. A computational approach may also better explain some of the divergence in our results, such as why better metacognition is associated with reduced iron in the visual cortex but increased iron in the precuneus and hippocampus. Furthermore, as brain myelination and iron are closely related to a variety of environmental and developmental factors, including stress and nutrition (Carlson et al., 2009, Sparkman and Johnson, 2008), future studies may greatly benefit from investigating the role of these variables in mediating the microstructural effects reported here.

Methodological limitations common to all studies requiring spatial normalisation are the potential for residual registration errors, as well as partial volume effects. To minimise these sources of bias we used the DARTEL algorithm for inter-subject registration, which results in maximally accurate registration (Klein et al., 2009), and used the voxel based quantification normalisation procedure to minimise partial volume effects introduced by smoothing (Draganski et al., 2011). Additionally, although here we report an effect of metacognition only in the left hippocampus, this laterality effect is likely due to a statistical thresholding effect. Indeed, both MT and R_2_* effects were observed in the right hippocampus at more liberal statistical thresholds.

Finally, in this study we used a VOI analysis on the basis of regions previously reported to show morphological covariation (Fleming et al., 2010b, McCurdy et al., 2013). Although we found myelination and iron effects in several of these areas (e.g., the dorsolateral/anterior PFC, precuneus), we failed to find significant effects in others (e.g., rostrolateral prefrontal cortex, superior parietal cortex). These differences raise interesting questions about the biological basis of these effects, and may suggest that the relationship between metacognition and neuroanatomy in DLPFC and precuneus is related to changes in underlying myeloarchitecture, rather than gray matter expansion. However, VBM studies are complicated by their dependence on tissue microstructure, algorithms used and the acquisition scheme which generates the image contrast driving the segmentation (Lorio et al., 2016, Lorio et al., 2014). Ultimately, the quantitative nature of the analyses presented here facilitate more direct replication testing in future investigations of the biological basis for metacognition.

## Conclusion

Here we use a quantitative multi-parameter mapping approach to reveal the relationship of the brain microstructure to perceptual metacognition. Importantly, as our method yields standardised metrics sensitive to the underlying tissue microstructure, these results can be used to inform future clinical research as they can be compared directly across research sites. Our results suggest that microstructure of the hippocampus is an important predictor for metacognitive ability. More generally, these results suggest that memory-related systems may be more important than previously realized for perceptual metacognition. Future research into the genetic, environmental, and other developmental factors underlying these findings are likely to yield strong dividends in the study of metacognitive ability.

## Figures and Tables

**Table 1:** Behavioural summary.

Variable	μ*S* (*SD*)	σ*S* (*SD*)	μ (μ*S,*σ*S)* (*SD*)
d’	1.40 (0.32)	1.60 (0.41)	−0.20 (0.43)
RT (ms)	404.3 (66)	414.0 (69)	409.1 (66)
Accuracy %	73.4 (3.6)	76.8 (4.9)	75.3 (3)
Confidence (1–4)	2.75 (0.26)	2.39 (0.27)	2.56 (0.14)
Confidence (1–100)	73.4 (13.8)	62.6 (15.2)	62.64 (15.23)
AROC	0.68 (0.07)	0.69 (0.08)	0.68 (0.06)
Median μ or σ	6.46 (2.96)	53.34 (13.27)	*na*

*Note:* μ*S* = mean staircase, σ*S* = variance staircase, *SD* = standard deviation.

**Table 2:** Summary of brain results.

VOI Results									
Map	Region	k	*p*_FWE_ peak	*p*_U_ peak	*t*	*z*	x	y	z
MT +	R aPFC	38	0.035	<0.001	4.38	3.92	37	41	22
MT -	R RSPC/Prec	25	0.024	<0.001	4.54	4.03	10	−58	8
R_2_* +	R Precuneus	51	0.040	<0.001	4.20	3.78	9	−64	24
R_1_ +	R aPFC	51	0.043	<0.001	4.25	3.82	37	42	22

Whole Brain Results									
Map	Region	k	*p*_FWE_ cluster	*p*_U_ cluster	*t*	*z*	x	y	z
MT -	L Hippo.	850	0.030	<0.001	5.48	4.68	−31	−25	−14
R_2_* -	L & R V1	598	0.016	<0.001	4.89	4.28	1	−69	11
R_2_* +	L MTG	568	0.011	<0.001	5.72	4.83	−51	−48	2
R_2_* +	R Subiculum^a^	417	0.044	<0.001	6.17	5.10	14	−38	−5
R_2_* +	L Hippo.^b^	2635	0.011	<0.001	4.94	4.31	−26	−36	−13

*Note:* Hippo = hippocampus, MTG = middle temporal gyrus, aPFC = rostrolateral prefrontal cortex, V1 = primary visual cortex, RSPC = Retrosplenial cortex.

+- indicates positive or negative t-constrast. See *VBQ analysis* and *VBQ Results* for more details.

a indicates peak-corrected result and p-values.

b indicates result from exploratory *p*<0.005 inclusion threshold. *k =* cluster extent in voxels, *t* = t-value, *z* = z-value, x,y,z = MNI peak coordinates. *p*FWE cluster = family-wise corrected cluster p-value, *p*U cluster = uncorrected cluster p-value, *p*FWE peak = family-wise corrected peak p-value, *p*U cluster = uncorrected peak p-value.
