# Supplementary material for: Metacognitive ability correlates with hippocampal and prefrontal microstructure
Source: Neuroimage. 2017 Apr 1;149:415–23. doi: 10.1016/j.neuroimage.2017.02.008 (PMC5387158; doi:10.1016/j.neuroimage.2017.02.008)
Supplement: Supplementary file 1 — Supplementary material [file mmc1.docx]

**SUPPLEMENTARY METHODS & RESULTS**

**Exploratory Analyses**

Given the novelty of linking hippocampal microstructure to metacognitive ability, we performed several post-hoc exploratory analyses, to inform future research on the microstructural underpinnings of metacognition. To this end we assessed 1) whether any of the hippocampus-metacognition correlation was explained by a measure of (auditory) memory capability, 2) whether brain iron in other cortical areas could explain this link, 3) whether any volumetric effects underpinned these or our other results, and 4) whether any informative effects could be found in the proton-density maps.

*Non-Verbal Auditory Memory Measure*

To explore the relationship between hippocampal microstructure and metacognition observed here (see Results), we collected a brief measure of participant’s non-verbal auditory memory using a previously validated same-different comparison paradigm (Müllensiefen et al., 2014; Harrison et al., 2016). This involved a short AB task in which participants listened to a sequence of 10-17 notes, and then decided if a subsequent sequence at a different absolute pitch level had the same or a different structure. The second note sequence was always transposed either by a fifth or by a semitone. Participants were required to indicate whether the two note sequences had an identical pitch interval structure or not. The test score was then calculated as the accuracy of the same-different judgement for each participant across all 13 trials.

*Mediation Analysis of Auditory Memory and Metacognition*

To explore the relationship of metacognition, auditory memory, and brain microstructure (see VBQ Results) we conducted a single-level mediation analysis (Wager et al., 2009; Woo et al., 2015). We chose to examine memory ability, as the function and structure of the hippocampus is closely related to memory in all sensory modalities (Squire, 1992; Zeidman and Maguire, 2016), and our VBQ analysis unexpectedly revealed striking microstructural correlations with metacognition in that region. As a 3-variable path model (Baron and Kenny, 1986), mediation analyses tests if the relationship between and input (X) and output variable (Y) is mediated by a third variable (M). It compares two models: a reduced model without mediation (eq., 1) and a full model with mediation (eq., 2):

$Y=cX+e_{y}$ (0.2)

$M=aX+e_{M}; Y=bM+c^{'}X+e_{Y}$ (0.3)

The test of meditation thus asks if the difference:

$$c-c^{'}=ab$$

is significantly different from zero. If so, it indicates that some portion of the variance in Y that is explained by X alone (equation 1) can be explained by the mediator variable M, leading to a reduced value of c’ (equation 2) compared to c (equation 1).

Here we asked whether the relationship of, e.g., hippocampal microstructure (Y) and memory (X) was mediated by individual differences in metacognitive ability (M). For this analysis, R2* and MT values were extracted from each subject within the peak coordinate identified by our multiple-regression analysis, for the aPFC (MT only), precuneus, and left hippocampus (see VBQ Results, below). As we observed a similar pattern of correlation for AROC in both hippocampus and precuneus/retrosplenial cortex, we first assessed the degree of correlation between each region for R2* and MT, respectively. Indeed, R2* and MT were significantly correlated between both regions (R2* between region *r*(df) = .37, *p* = .009; MT between region *r*(df) = .46, *p* = .002). We thus averaged the values of each parameter across regions to create a general hippocampal-precuneus (HP) index for both measures. We then fit single-level mediation analyses using the Multilevel Mediation and Moderation Toolbox for MATLAB (https://github.com/canlab/MediationToolbox) to HP R2*, HP MT, and aPFC MT while controlling for the same nuisance covariates as in our VBQ analysis. Group-level significance for parameters of the path-model (e.g., ab) was estimated using bias-corrected bootstrap testing (Efron and Tibshirani, 1994) with 10,000 samples.

*Whole Brain Mediation Analysis*

Because brain iron and myelination interact in a complex process of iron homeostasis, we were interested to explore whether our link between hippocampal MT and metacognition could be explained by changes in iron elsewhere in the brain. To investigate this, we ran another mediation analysis similar to the above, but this time searching through the entire R2* map for voxels which showed a significant mediation effect. This analysis was controlled for age, gender, and total intracranial volume and was also estimated using bias-corrected bootstrap testing with 10,000 samples. This yielded p-value maps for the *a* path parameter (AROC - > whole brain iron), *b* path parameter (whole-brain iron - > hippocampal MT), and *ab* parameter (iron mediation effect). Statistical significance was assessed using whole brain FDR correction, *p* < .05. For visualization purposes, effects were plotted at *p* < .001 uncorrected on the average MT map (see Supplementary Figure 5).

*Voxel-Based Morphometry Analysis*

To assess whether the microstructural effects observed here where related to previously reported volumetric effects, we performed a voxel-based morphometry (VBM) analysis using SPM12 at the request of a reviewer. To do so we created ‘synthetic’ MPRAGE-like images by recombining our quantitative images into a single map. For comparison, we also performed a VBM analysis using our MT-derived segmentations.

**Image pre-processing: creation of synthetic MPRAGE-like images**

**Image synthesis:** First, two synthetic FLASH volumes were created using the FreeSurfer mri_synthesize routine. Inputs to the routine were scaled quantitative PD and T_1_ (1/R_1_ volumes), with removal of a small number of negative and very high values produced by estimation errors. For the first synthetic image, default FreeSurfer contrast parameters were specified. The second synthetic image was produced using the same PD and T1 input volumes, with contrast parameters specified (TR = 25 ms; α = 220°; TE = 4 ms). During synthesis, images were ‘conformed’ to 1mm^3^ isotropic resolution in FreeSurfer. Both synthetic images were then further scaled with AFNI 3dcalc; this additional linear scaling yielded image intensity properties closer to the optimal intensity values needed to segment tissue boundaries in FreeSurfer. The image synthesized with default contrast parameters was used as the main input to the FreeSurfer automated processing stream (following further pre-processing steps; see below). The image synthesized with specified contrast parameters was used at a later stage as input to the FreeSurfer Talairach transformation. Finally, a scaled and truncated version of the PD volume was produced with AFNI 3dcalc. This adjusted PD volume was used as input to the skull strip procedure (see below).

**Manual image adjustment:** Each subject’s synthetic image was hand-adjusted using a piecewise linear normalization procedure to linearly ramp intensity values of grey and white matter within isolated regions. Brightness values of voxels within the inferior and medial temporal lobes, temporal pole, long and short insular gyri, and ventro-medial pre-frontal cortex were gently rescaled (< 1.2x). Manual blink comparison between the synthetic volume and the labelled white matter surface was used to compare adjustments as each brightening iteration was applied. Care was taken to ensure that manual brightening did not cause grey and white matter to exceed the intensity value bounds specified for those tissue classes in FreeSurfer (grey matter: 50-70; white matter: 100-140). Manually brightened synthetic images were saved and used within the skull strip procedure.

**Skull strip**: Next, the subject’s adjusted quantitative PD volume (see image synthesis) was used as input to a customized skull strip procedure run in Csurf. Briefly, the skull strip procedure removed the skull and regions exterior to it from the image volume, rendering an image of remaining brain tissue (including cerebellum and brainstem). First, an elliptical surface (4th or 5th geodesic subtessellation of an icosahedron) was expanded from inside the PD volume, with expansion of the surface constrained by arrival at low intensity voxels (i.e., those containing CSF and/or the inner surface of the skull). The set of voxels intersecting the faces of the resulting surface was then flood-filled from the outside, thereby constraining the brain volume to the brighter voxels inside the surface region. Using this PD volume as a mask, flood-filled voxels in the volume were used to set the corresponding voxels in the subject’s default-parameter synthetic image to an intensity of zero. The boundaries of the flood-filled voxels within the skull-stripped PD image were then manually adjusted to correct for any local deviations into neural tissue (particularly in regions proximal to paranasal sinuses, prone to susceptibility artefacts). Manual adjustment involved reducing the intensity threshold for cortical grey matter (to a value of 40); the flood-filled boundary was then forced toward voxels below this threshold. Manual adjustment was applied to the synthetic volume; the skull-stripped synthetic volume was used as input

Following similar preprocessing protocols as used by Fleming et al. (2010) and McCurdy et al (2013), the synthetic MPRAGE-like images were then segmented into gray matter, white matter, and CSF in native space. The DARTEL (diffeomorphic anatomical registration through exponentiated lie algebra) algorithm (Ashburner, 2007) was used for normalization to increase the accuracy of inter-subject registration, by aligning and warping the gray matter images to an iteratively improved template. The DARTEL template was then registered to the Montreal Neurological Institute stereotactic space, and the gray matter images modulated such that their original tissue volumes were preserved. Images were smoothed using an 8mm full-width at half-maximum Gaussian kernel. The resultant pre-processed images were then analysed in a multiple regression design matrix to identify brain regions whose volume correlated with metacognitive sensitivity (AROC). As in our VBQ analyses and in previous investigations, participant’s noise-induced confidence bias, average confidence, response bias, motion d-prime, mean signal difference, age, and gender were included as control covariates. Proportional scaling was used to account for variability in total intracranial volume across participants. A binary gray matter mask (> 0.3) was generated from the average of all subjects normalized, smoothed gray matter segments, to exclude clusters outside the brain and limit the search volume to voxels likely to contain gray matter.

We examined the positive and negative t-maps for the AROC effect, in both a whole brain FWE-cluster corrected (non-stationarity corrected, inclusion threshold *p* < .001) analysis and a FWE-peak corrected VOI analysis using the same mask of a priori regions as in our VBQ analysis, which was generated by placing a 5mm sphere at the peak voxel coordinates reported by Fleming et al. (2010) and McCurdy et al (2013). This procedure was also repeated using an identical preprocessing and analysis, but for the MT-derived segmentations.

*Proton Density VBQ*

At the request of a reviewer, we also analysed the effective proton-density maps produced by the MPM protocol. Due to averaging across the first six echoes of the FLASH volumes, this measure has an effective echo time of 8 ms leading to some residual T2* weighting, such that the PD would be underestimated in regions of high iron. The PD map thus provides an estimate of the mobile proton density (i.e., free water) (Tofts, 2005; Helms et al., 2009; Callaghan et al., 2014; Lorio et al., 2014). We used the same approach as in our overall VBQ analyses, examining both effects within the a priori VOI mask and whole-brain cluster level effects.

**Supplementary Results**

*Mediation Results - Auditory Memory and Metacognition*

This analysis revealed a significant relationship between memory scores and AROC (*M* path = 0.39, *SD* = 0.17, *p* < .05). Across all three brain measures, AROC significantly mediated the relationship of brain microstructure and memory ability – for MT this relationship was positive in the aPFC, with higher metacognition mediating the relationship of memory and aPFC (*M* path = 0.24, *SD* = 0.15, *p* < .05); instead suppression (negative mediation) was observed for hippocampal-precuneus (*M* path = -0.34, *SD* = 0.16, *p* < .05). Finally, for HP R2* AROC also showed a positive mediation effect (*M* path = 0.27, *SD* = 0.16, *p* < .05). These results suggest that metacognition differentially changes the interrelation of prefrontal and hippocampal microstructure to memory ability, indicating that individuals who are better at metacognition have a more metacognition-related hippocampus and conversely, a more auditory memory-related prefrontal cortex. See Supplementary Figure 4 for an overview of these results.

*Mediation Results – Whole Brain Iron Mediation*

For the *ab* path, this analysis revealed a significant suppression effect (negative mediation) in the precuneus and ventromedial prefrontal cortex, which did not survive FDR correction for multiple comparisons. Comparison of the direct (*c*) vs indirect (*c’*, i.e., after mediation) effects suggested that accounting for iron in the precuneus reduced the correlation of hippocampal MT and metacognition from *r*(df) = -0.59 to -0.35. Inspection of the *a* path revealed several brain areas in which metacognition showed significant FDR-corrected positive correlations with iron including the precuneus, bilateral hippocampus, insula, and superior parietal cortices (see supplementary table 1), whereas the b-path showed negative correlations of precuneus iron level and hippocampal MT (Supplementary Figure 5D). These results suggest that iron levels in the midline and hippocampal cortices are related to hippocampal myelination and metacognitive ability.

*VBM Results*

Although significant correlations were observed in cortical areas previously implicated in metacognitive sensitivity, including the right anterior PFC and right posterior cingulate (see Supplementary Figure 1), these did not survive either peak or cluster-level correction for multiple comparisons. Additionally, no significant effects were found within the VOIs reported by previous studies, although one correlation in the right APFC was within 10mm of the coordinates reported by Fleming et al. (2010) and McCurdy et al (2013). No negative correlations were observed.

This analysis indeed suggests partial evidence for the reproducibility of these volumetric effects; however it should be noted that comparing VBM results across studies is inherently difficult due to the dependency of volumetric measures on scanner and acquisition protocols. In particular, although we attempted to match our preprocessing and analysis protocol with previous studies, our reliance on MPRAGE-like synthetic volumes further complicates this comparison.

Finally, at a reviewer’s request we also performed an identical analyses using our MT-derived segmentations. This analysis demonstrated similar results as to our synthetic MPRAGE-like analysis. Overall the spatial topology of correlations was largely overlapping the MPRAGE-like analysis. See supplementary figure 2 for an illustration and comparison of these results.

*Proton Density VBQ Results*

Although we observed an (uncorrected) significant positive correlation in the right rostrolateral prefrontal cortex (Supplementary Figure 3), no effects survived multiple comparison correction. Our VOI analysis also observed a negative correlation in the same right APFC coordinate where we found significant positive effects of MT and R1, but this did not survive small-volume correction (*p*FWE = 0.151, *t* = 3.54).

All uncorrected supplementary t-maps are available at our Neurovault collection: (<http://neurovault.org/collections/1260/>).

**Supplementary Figures**


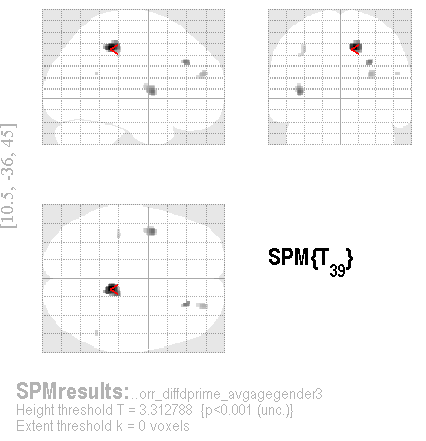

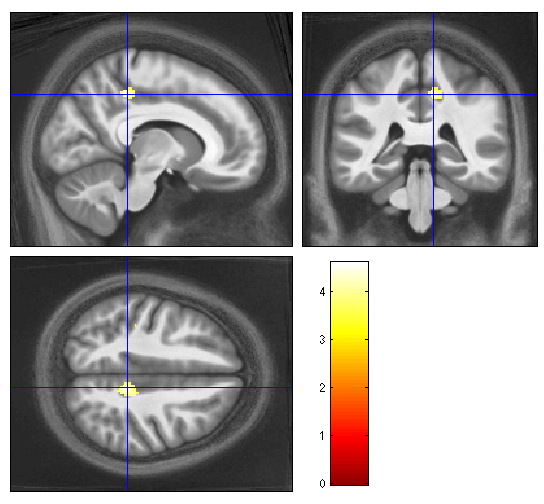

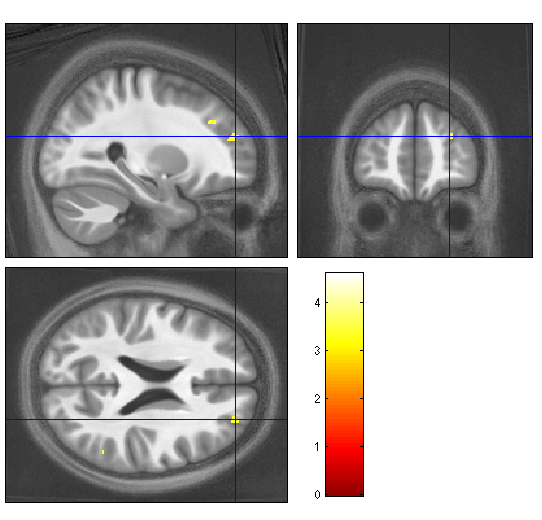


**Supplementary Figure 1: VBM analysis**. At an uncorrected level, positive correlations in the posterior cingulate, left insula, and right anterior prefrontal cortex/DLPFC can be observed. However no effects survived correction at either the whole-brain or VOI level. Note that the right APFC effect is within 10 mm of the coordinates reported by Fleming et al. (2010) and McCurdy et al (2013).


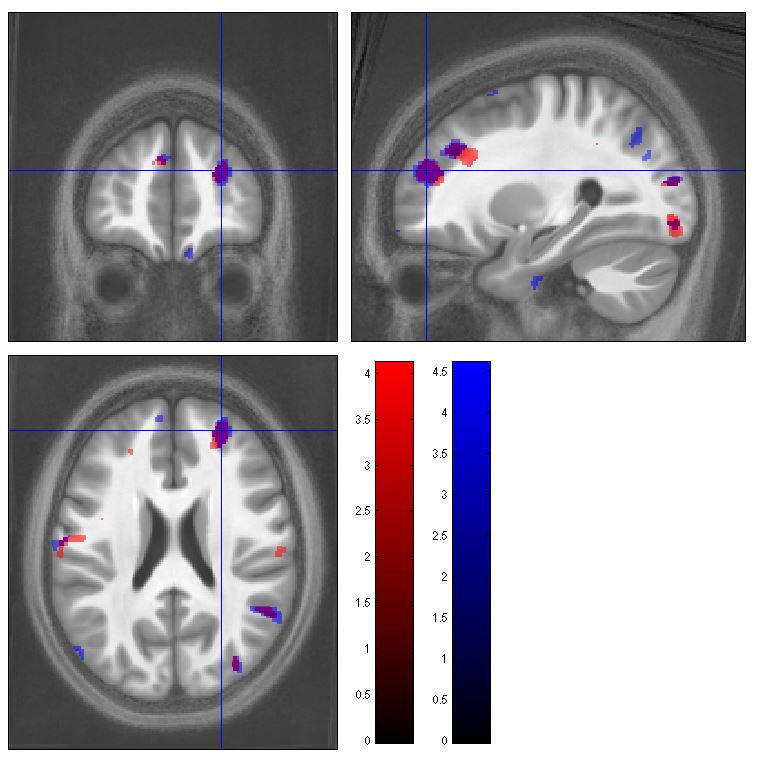

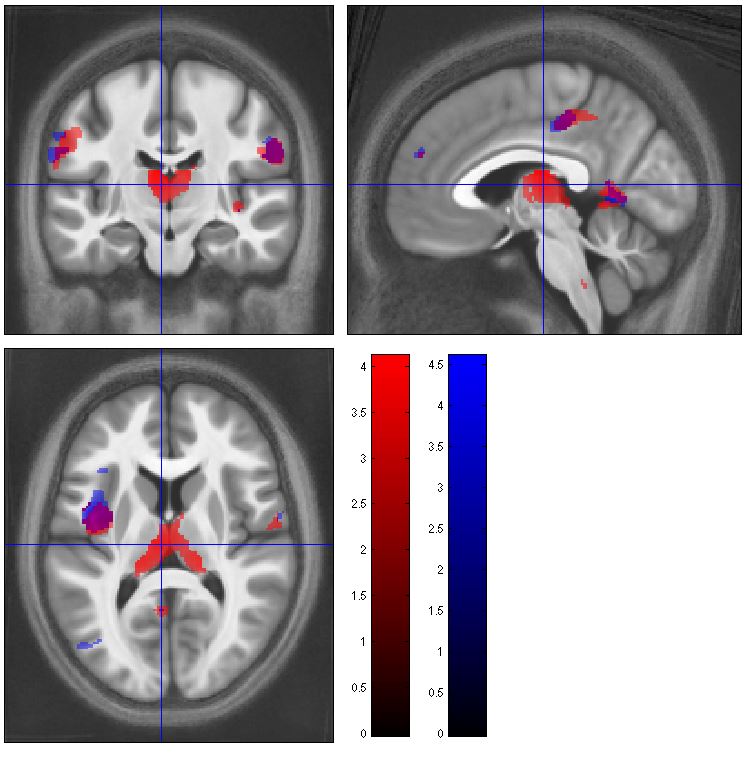


**Supplementary Figure 2: VBM analysis comparing synthetic MPRAGE (blue) vs MT-segmentation derived (red) results**. The two methods show highly similar topology. At an uncorrected level, positive correlations in the posterior cingulate, left insula, and right anterior prefrontal cortex/DLPFC can be observed in both maps. Note that no effects survived correction at either the whole-brain or VOI level. Colorbars depict t-value at each voxel. Cross-coordinates MNI xyz = -4, -18, 10 (right panel), and MNI xyz = 27, 48, 19 (left panel).


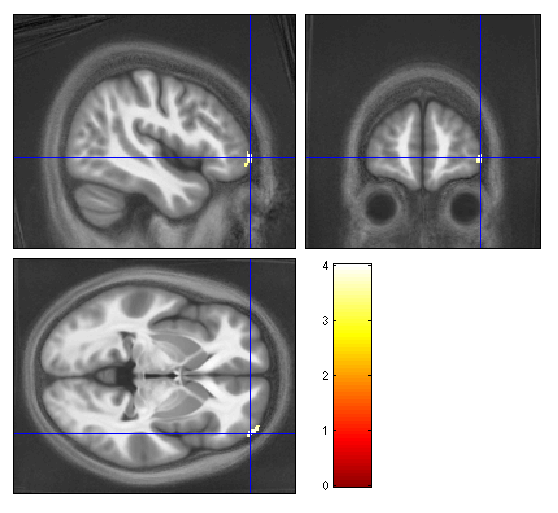


**Supplementary Figure 3: VBQ analysis of effective proton density map**. A significant positive correlation with AROC is observed at an uncorrected level in the right rostrolateral PFC. No effects surviving multiple comparisons correction were observed. Colorbar depicts t-map.

**
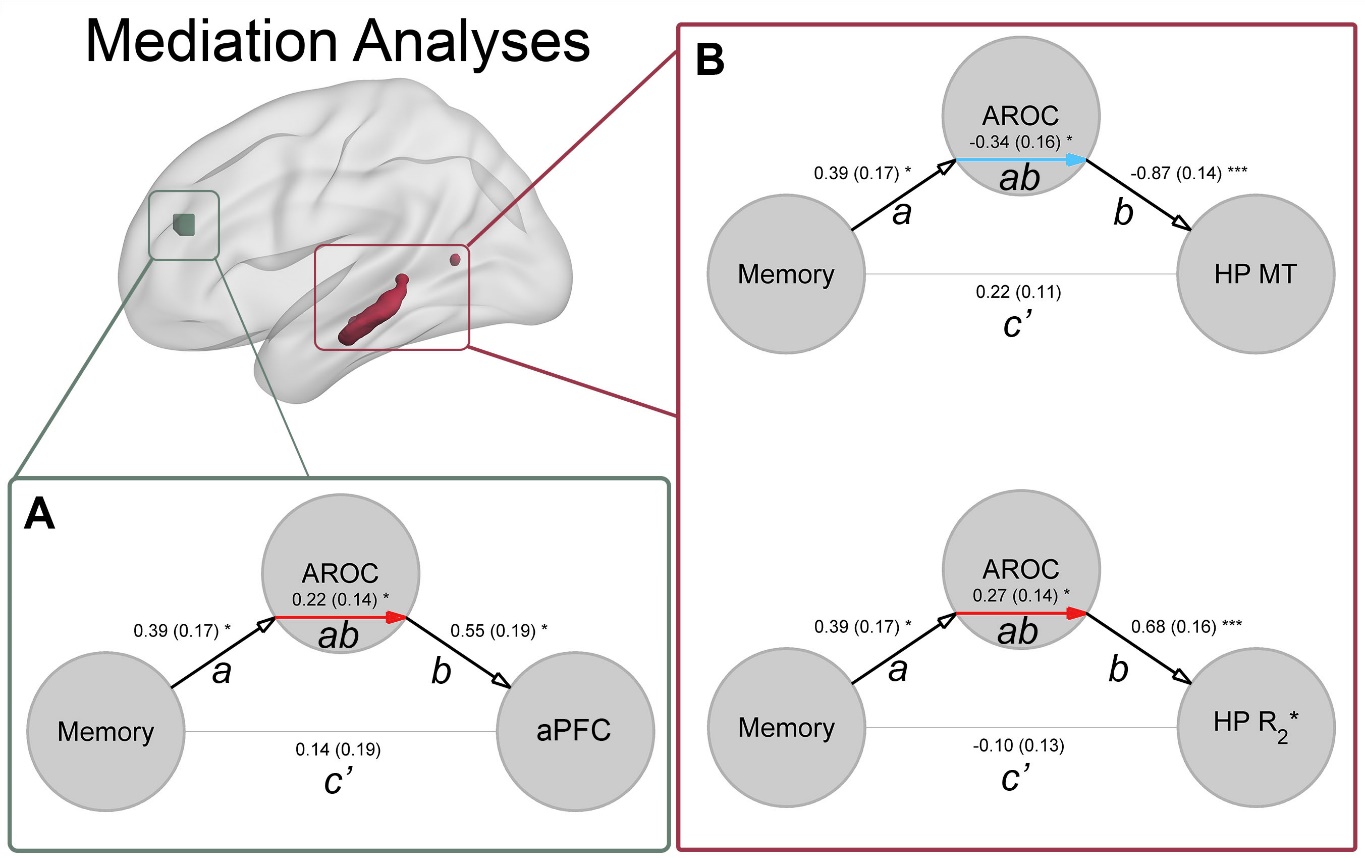
**

**Supplementary Figure 4, Auditory Memory and Metacognition Mediation Analysis Results. A)** Metacognitive ability (AROC) significantly mediates (red arrow) the relationship of non-verbal auditory memory and aPFC myelo-architecture (as measured by Magnetization Transfer Ratio, MT). In contrast, **B)** the relationship of memory and hippocampal-precuneus/retrosplenial cortex (HP) myelo-architecture is supressed by metacognitive ability (blue arrow), but enhanced for HP iron (as measured by R_2_*). These results suggest individual differences in memory and metacognition are related by a dynamic interaction between the two networks, with the memory-related network increasingly related to metacognitive circuits and vice versa for the prefrontal cortex. Statistical significance for path model parameters determined via bootstrapping procedure, see *Supplementary Methods* for more details (* < .05, *** < .001).


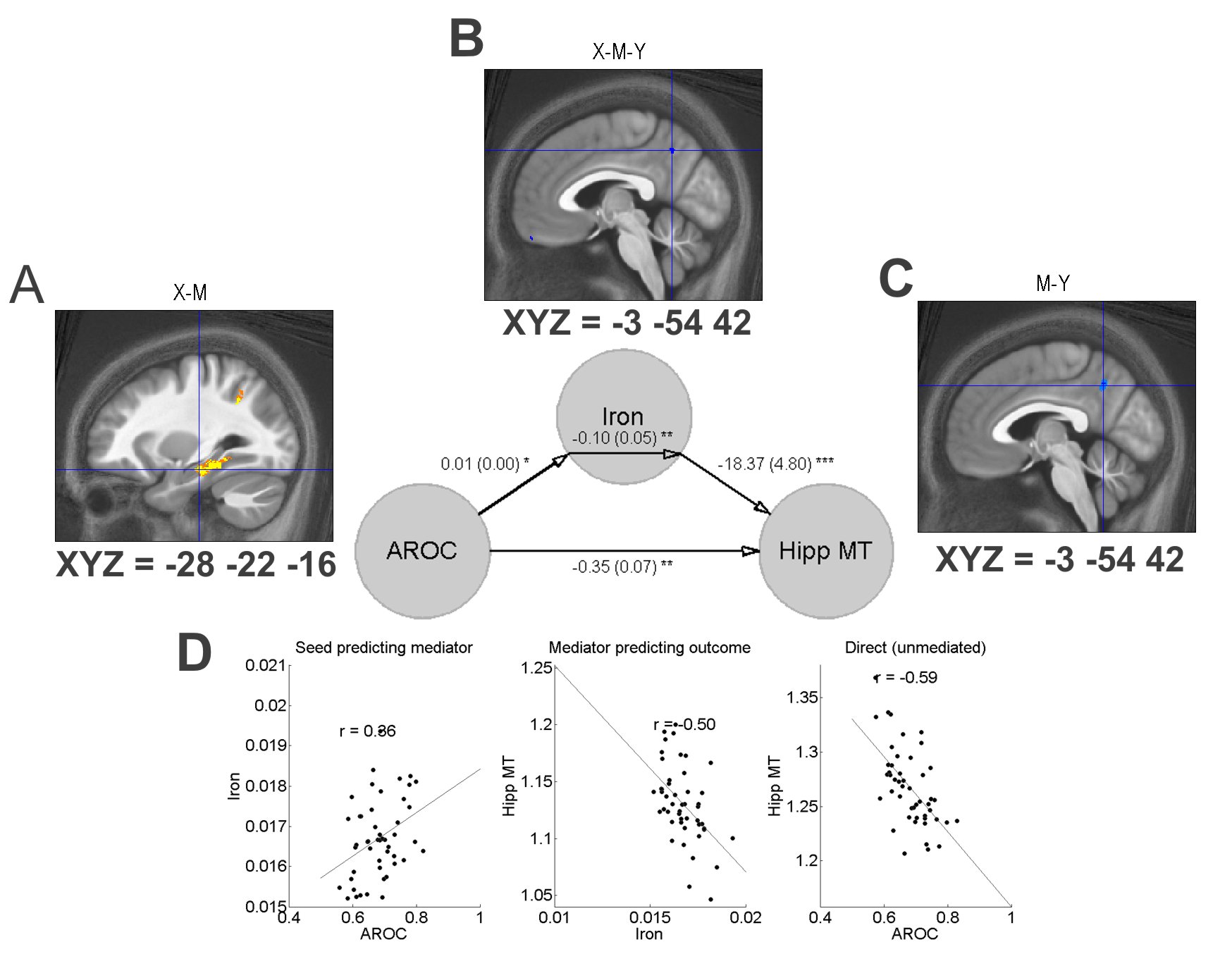


**Supplementary Figure 5,** Whole-brain R_2_* mediation analysis. Figure shows results of whole-brain mediation analysis, in which our brain iron marker (R_2_*) mediated the relationship of metacognition (AROC) and hippocampal myeloarchitecture (MT). **A**) plot shows significant (FDR corrected *p* < .05) positive correlation of AROC and iron (*a* parameter in the mediation analysis) in the left hippocampus and precuneus (not shown). **B**) Significant (*p* < .001 uncorrected) suppression (i.e., negative mediation, *ab* parameter) of the AROC-MT relationship by iron levels in the precuneus and vMPFC. **C)** Significant (FDR corrected *p* < .05) negative correlation of precuneus iron and hippocampal MT (*b* parameter). **D)** Scatterplots illustrating mediation model effects for extracted precuneus coordinate [MNI = -3, -54, 42]; mediation effect of precuneus iron reduces the AROC-Hippocampal MT effect from *r*(df) = -0.59 to -0.35 (bottom left panel vs *c*’ parameter in path diagram). These results suggest that in individuals with higher levels of iron in the midline cortices, metacognitive ability is less related to hippocampal myeloarchitecture.

**Supplementary References**

Ashburner J (2007) A fast diffeomorphic image registration algorithm. NeuroImage 38:95–113.

Baron RM, Kenny DA (1986) The moderator–mediator variable distinction in social psychological research: Conceptual, strategic, and statistical considerations. J Pers Soc Psychol 51:1173.

Callaghan MF, Freund P, Draganski B, Anderson E, Cappelletti M, Chowdhury R, Diedrichsen J, FitzGerald THB, Smittenaar P, Helms G, Lutti A, Weiskopf N (2014) Widespread age-related differences in the human brain microstructure revealed by quantitative magnetic resonance imaging. Neurobiol Aging 35:1862–1872.

Efron B, Tibshirani RJ (1994) An introduction to the bootstrap. CRC press.

Fleming SM, Weil RS, Nagy Z, Dolan RJ, Rees G (2010) Relating Introspective Accuracy to Individual Differences in Brain Structure. Science 329:1541–1543.

Harrison PMC, Musil JJ, Müllensiefen D (2016) Modelling Melodic Discrimination Tests: Descriptive and Explanatory Approaches. J New Music Res 45:265–280.

Helms G, Draganski B, Frackowiak R, Ashburner J, Weiskopf N (2009) Improved segmentation of deep brain grey matter structures using magnetization transfer (MT) parameter maps. NeuroImage 47:194–198.

Lorio S, Lutti A, Kherif F, Ruef A, Dukart J, Chowdhury R, Frackowiak RS, Ashburner J, Helms G, Weiskopf N, Draganski B (2014) Disentangling in vivo the effects of iron content and atrophy on the ageing human brain. NeuroImage 103:280–289.

McCurdy LY, Maniscalco B, Metcalfe J, Liu KY, Lange FP de, Lau H (2013) Anatomical Coupling between Distinct Metacognitive Systems for Memory and Visual Perception. J Neurosci 33:1897–1906.

Müllensiefen D, Gingras B, Musil J, Stewart L (2014) The Musicality of Non-Musicians: An Index for Assessing Musical Sophistication in the General Population. PLOS ONE 9:e89642.

Squire LR (1992) Memory and the hippocampus: A synthesis from findings with rats, monkeys, and humans. Psychol Rev 99:195–231.

Tofts P (2005) Quantitative MRI of the brain: measuring changes caused by disease. John Wiley & Sons.

Wager TD, van Ast VA, Hughes BL, Davidson ML, Lindquist MA, Ochsner KN (2009) Brain mediators of cardiovascular responses to social threat, Part II: Prefrontal-subcortical pathways and relationship with anxiety. NeuroImage 47:836–851.

Woo C-W, Roy M, Buhle JT, Wager TD (2015) Distinct Brain Systems Mediate the Effects of Nociceptive Input and Self-Regulation on Pain. PLOS Biol 13:e1002036.

Zeidman P, Maguire EA (2016) Anterior hippocampus: the anatomy of perception, imagination and episodic memory. Nat Rev Neurosci 17:173–182.
